# Cardiovascular Complication following Covishield Vaccination in Nepal: A Case Report

**DOI:** 10.31729/jnma.6454

**Published:** 2021-08-31

**Authors:** Angela Basnet, Shiva Kumar Ojha, Suman Kumar Jha, Anjana Paudyal, Manoj Khadka, Manita Khadka, Dhan Bahadur Shrestha

**Affiliations:** 1 Department of Internal Medicine, Scheer Memorial Adventist Hospital, Banepa, Kavrepalanchok, Nepal; 2 Nepalese Army Institute of Health Sciences, Sanobharyang, Kathmandu, Nepal; 3 Department of Internal Medicine, Mount Sinai Hospital, Chicago, USA

**Keywords:** *bundle-branch block*, *case report*, *COVID-19 vaccine*, *Nepal*

## Abstract

Nepal started the COVID-19 vaccination on 27 January 2021 with AstraZeneca/Oxford Coronavirus Disease-19 AZD1222 (Covishield) vaccine to control the Coronavirus disease pandemic. The vaccine has a good safety profile, with cardiovascular complications being rare. Herein we report a rare case of cardiovascular complication following Covishield vaccination in a 33 years old female who had dizziness and elevated blood pressure immediately following vaccination and abnormal electrocardiogram showing T wave inversions followed by left bundle branch block. The patient was kept on observation, following which the blood pressure and electrocardiogram changes were normal by seven days. This cardiovascular complication following the vaccination demands further investigation into the adverse event of the vaccine. However, since the benefit of the vaccine outweighs the risk, World Health Organization has recommended the continuity of the vaccine as of now.

## INTRODUCTION

World Health Organization (WHO) declared Coronavirus disease (COVID-19) a pandemic on 11 March 2020.^1^ To control this global issue, the first mass vaccination program for COVID-19 started in early December 2020.^2^ It was on 27 January 2021 when Nepal initiated the COVID-19 vaccination with AstraZeneca/Oxford COVID-19 AZD1222 (Covishield) vaccine.^3^ The commonly reported side effects were pain at the site of injection, myalgia, headache, nausea, and fever.^3^ Cardiovascular complications are rarely reported. Herein we report a rare case of cardiovascular complication following Covishield vaccination in Nepal, which demands the need for further investigation into the adverse event of the vaccine.

## CASE REPORT

A 33 years old female, who received the Covishield vaccination at Scheer Memorial Adventist Hospital, Banepa on 28 January 2021, immediately following the vaccination had dizziness, generalized discomfort, and the urge to leave the observation room due to the feeling of lack of air in the room. Her blood pressure recorded was 160/110 mmHg and 180/130 mmHg immediately and after 20 minutes respectively. The pulse rate was 100/minute, respiratory rate of 20/minute, and normal temperature. The patient was kept in observation and consulted for cardiology and no intervention was done.

**Figure 1:**
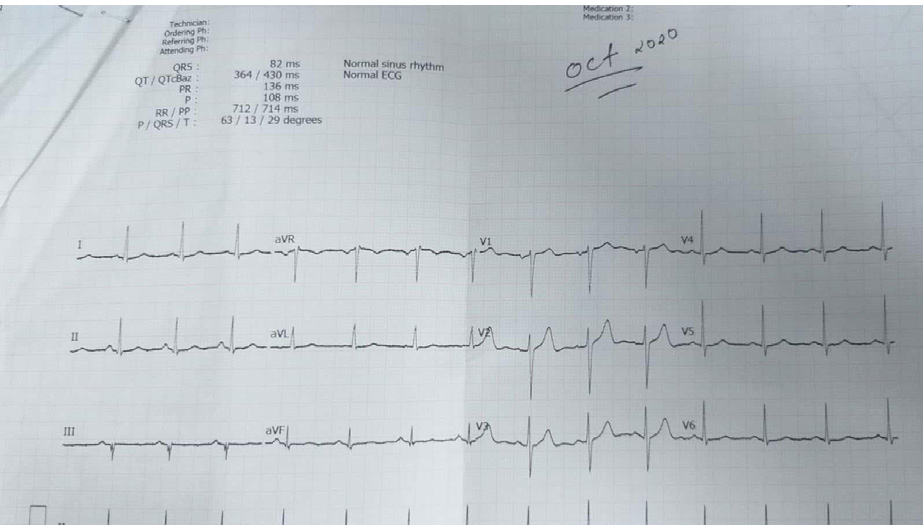
Normal ECG showing normal sinus rhythm in October 2020 (before vaccination).

There was no significant past medical history except hypertriglyceridemia for which she was under regular medicine. There was no significant family and psychosocial history. There was no history of high blood pressure and an electrocardiogram (ECG) showed normal sinus rhythm in the past (Figure 1).

**Figure 2:**
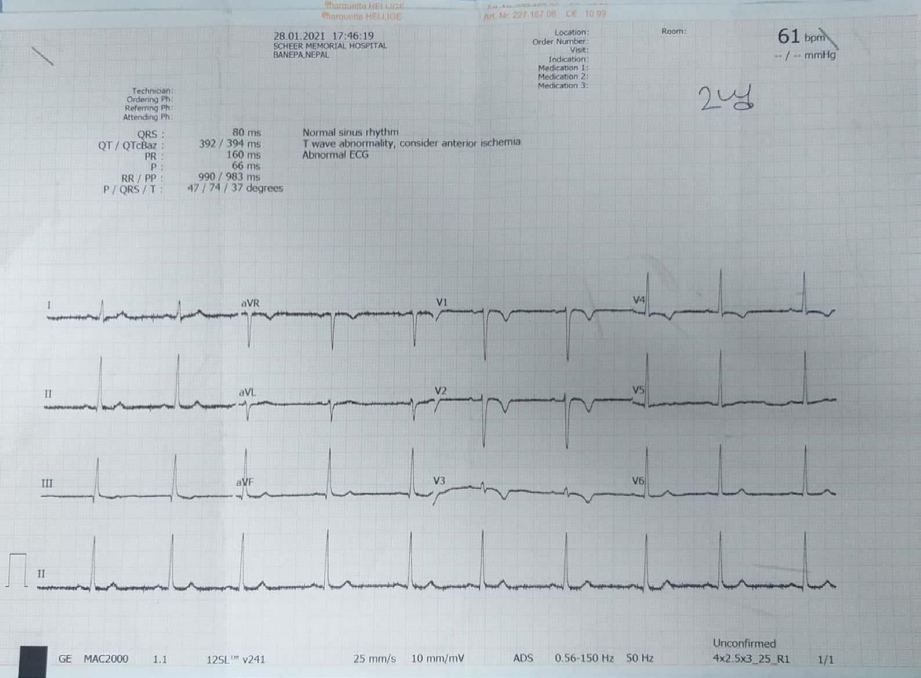
Abnormal ECG showing T wave abnormality on 28 January 2020.

**Figure 3:**
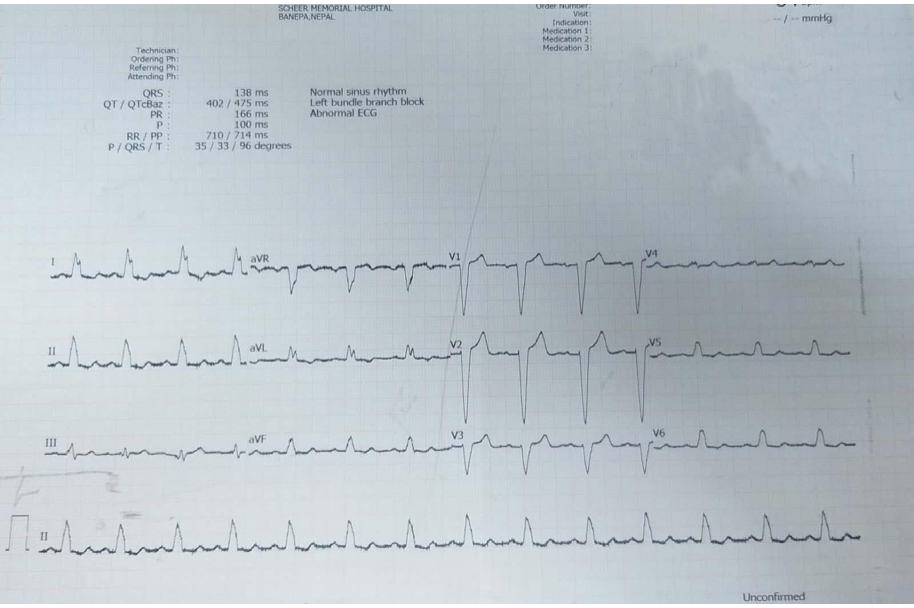
Abnormal ECG showing left bundle branch block on 29 January 2021.

An ECG done on 28 January 2021 showed T wave inversions in leads V1, V2, V3, and V4 (Figure 2) followed by new changes of the left bundle branch block the next day (Figure 3). Cardiac enzymes were sent with troponin I being negative and creatine kinase (CK-MB) within the normal range. The blood pressure continued to stay elevated for the next 24 hours following which it gradually normalized by day 7. The ECG changes remained the same for the next 7 days and repeat ECG after 7 days showed normal sinus rhythm with normal QRS complexes.

## DISCUSSION

Coronavirus was named so because of the crownlike appearance in the electron microscope, which is attributed to the spike glycoprotein that radiates from the viral surface like a solar corona.^4^ The spike glycoprotein (S) consists of two subunits, the SI subunit helping the virus bind with the Angiotensin-Converting Enzyme-2 (ACE2) receptor, present on the surface of type 2 pneumocyte in lung and S2 subunit helping in cellular fusion.^4,5^ So, targeting spike glycoprotein can neutralize the infection by the virus.

Many COVID-19 vaccines are using spike glycoprotein as the target antigen.^5^ One of them being Oxford / AstraZeneca COVID-19 AZD1222 (Covishied) vaccine, which contains the non-replicating simian adenovirus vector with SARS-CoV-2 spike protein in it.^5^ The vaccine is administered in two doses and has a good safety profile.

A phase 1/2, participant-blinded, multicentre, randomised controlled trial done at five centres in the UK demonstrated local and systemic reactions like pain, muscle ache, headache, feeling feverish, chills, and malaise were more common in the AZD1222 vaccine group while no serious adverse events related to the vaccine were reported.^6^ An interim analysis of four randomised controlled trials in Brazil, South Africa, and the UK, which evaluated the safety and efficacy of AZD1222 vaccine against SARS-CoV-2 showed a good safety profile of the vaccine and serious adverse events (total of 175 events in 168 participants) balanced across both groups (84 events in 79 participants of vaccine group).^7^ Among 79 participants reporting serious adverse events in the vaccine group, five mentioned cardiac disorders (three angina pectoris, one atrial flutter, and one complete atrioventricular block) and none reported having vascular disorders.^7^ There are also reports of rare blood coagulation disorders in AstraZeneca COVID-19 vaccine recipients.^8^

Cardiovascular complications are rarely reported following the Covishield vaccination. Although cardiovascular complications following vaccination are rare, there are reports of myocarditis, pericarditis, and arrhythmias after smallpox vaccination back in the 1950s in the United States.^9^ Similarly in 188090s, seven cases of pericarditis were reported after Hepatitis B and influenza vaccination, while a case of myopericarditis was reported in 2000 following diphtheria, polio, and tetanus vaccination.^9^

Adverse events following vaccination campaigns have been quite a routine job and its reporting is very important to build up evidence on the safety of a vaccine. But this doesn't imply that all adverse events are related to the vaccine itself. Various factors like the patient's comorbid conditions, and immunity might also play a role. This case report doesn't imply the discontinuity of the vaccine since the benefit of the vaccine outweighs its risk. However, a deeper investigation into the adverse event of the vaccine is necessary.
